# Current and Future Perspectives of Cell-Free DNA in Liquid Biopsy

**DOI:** 10.3390/cimb44060184

**Published:** 2022-06-10

**Authors:** Shicai Liu, Jinke Wang

**Affiliations:** State Key Laboratory of Bioelectronics, Southeast University, Nanjing 210096, China

**Keywords:** liquid biopsy, cancer, cell-free DNA, next-generation sequencing, cancer detection

## Abstract

A liquid biopsy is a minimally invasive or non-invasive method to analyze a range of tumor material in blood or other body fluids, including circulating tumor cells (CTCs), cell-free DNA (cfDNA), messenger RNA (mRNA), microRNA (miRNA), and exosomes, which is a very promising technology. Among these cancer biomarkers, plasma cfDNA is the most widely used in clinical practice. Compared with a tissue biopsy of traditional cancer diagnosis, in assessing tumor heterogeneity, a liquid biopsy is more reliable because all tumor sites release cfDNA into the blood. Therefore, a cfDNA liquid biopsy is less invasive and comprehensive. Moreover, the development of next-generation sequencing technology makes cfDNA sequencing more sensitive than a tissue biopsy, with higher clinical applicability and wider application. In this publication, we aim to review the latest perspectives of cfDNA liquid biopsy clinical significance and application in cancer diagnosis, treatment, and prognosis. We introduce the sequencing techniques and challenges of cfDNA detection, analysis, and clinical applications, and discuss future research directions.

## 1. Introduction

Cancer is a major public health problem worldwide. It has become one of the most common diseases and frequently-occurring diseases, which causes the most harm to human health and seriously affects quality of life [1]. Cancer can be cured; the key is the “three early” factors. A lot of clinical practice has proved that some cancers can be cured by early detection, early diagnosis and early treatment [2,3,4]. If it reaches the late stage, modern medicine has no way to cure it. Therefore, the treatment of cancer should follow the prevention-based policy to achieve early detection, early diagnosis and to provide a reliable basis for the treatment of cancer, which is an important factor to reduce the death rate.

Although tissue biopsy is the most widely used method for diagnosis and prognosis of cancer [5], there are many shortcomings. A tissue biopsy requires tissue sampling, which can be traumatic, easily cause other complications, and the cost of sampling is very expensive. For instance, the study by Overman et al. showed that the rate of adverse events in lung cancer patients undergoing an intrathoracic image-guided biopsy was 17.1% (36 of 211 biopsies) [6]. In addition, when the tumor has not yet formed, it is not practical to use tissue biopsy for cancerearly detection/diagnosis. At present, there are some screening methods that have been proven to be effective for cancer prevention. For instance, a pap test, which detects changes in the level of cells in the cervix, is the first test to be used for cancer screening [7]. Low-dose computed tomography (LDCT) can effectively reduce the mortality of lung cancer and improve the prognosis [8]. Endoscopic screening can reduce the risk of death from esophageal cancer (ESCA) [9]. Fecal occult blood test (FOBT) can effectively reduce the mortality of colorectal cancer (CRC) [10]. Mammography screening can reduce breast cancer mortality [11]. However, all of these screening methods are applicable only to specific cancers with low sensitivity and specificity. In the large-scale cancer detection and screening of population, cancer detection and screening method with high flexibility and low cost is urgently needed.

**Figure 1:**
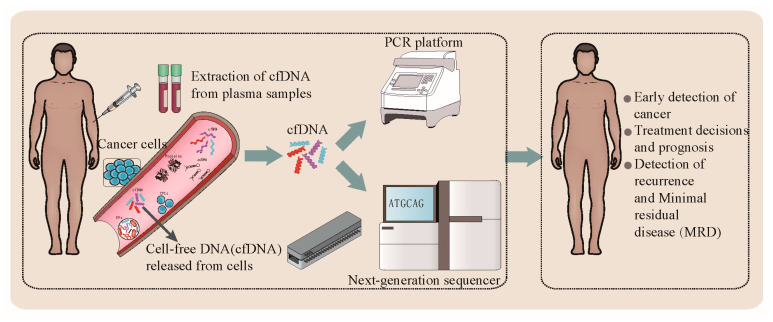
Clinical application of cell-free DNA (cfDNA) as liquid biopsy material.

The global liquid biopsy industry is expected to exceed USD 5 billion by 2023 [12]. A liquid biopsy is a minimally invasive or non-invasive method to analyze a range of tumor material in blood or other body fluids, including circulating tumor cells (CTCs), cell-free DNA (cfDNA), messenger RNA (mRNA), microRNA (miRNA), and exosomes, which is a very promising technology [13,14]. Among these cancer biomarkers, plasma cfDNA is the most widely used in clinical practice [15,16,17,18]. CfDNA released from tumors is also called circulating tumor DNA (ctDNA). CfDNA was first reported by Mandel and Metais in 1948 [19]. Leon et al. described cfDNA for the first time in the field of oncology, reporting cfDNA levels higher in cancer patients than in healthy individuals [20]. CfDNA comprises extracellular DNA molecules released into the blood through different mechanisms, including apoptosis, necrosis, senescence and active secretions [21,22]. Compared with tissue biopsy of traditional cancer diagnosis, in assessing tumor heterogeneity, a liquid biopsy is more reliable because all tumor sites release cfDNA into the blood. Therefore, a cfDNA liquid biopsy is less invasive and comprehensive. Moreover, the development of next-generation sequencing (NGS) technology makes cfDNA sequencing more sensitive than a tissue biopsy, with higher clinical applicability and wider application (Table 1). In this publication, we review the latest perspectives of cfDNA liquid biopsy clinical significance and application in cancer diagnosis, treatment, and prognosis. We introduce the sequencing techniques and challenges of cfDNA detection, analysis, and clinical applications, and discuss future research directions (Figure 1).

## 2. Potential and Applications

### 2.1. Early Detection

Early and effective diagnosis is considered essential in cancer disease, because early discovery can allow medical staff to treat patients earlier and more effectively, thereby greatly improving the survival rate of patients. Although early diagnosis of cancer has been studied around the world for many years, it is still a difficult task to better excavate cancer biomarkers with high sensitivity and specificity.

At present, compared with carcinogenic antigens, several cfDNA detection methods have been able to obtain higher sensitivity and specificity [45]. Phallen et al. found that cfDNA can be used for early lung cancer detection by directly evaluating the sequence change in cfDNA with ultra-sensitive evaluation [33]. In another prospective study, Gormally et al. found that two years before the cancer was diagnosed, *KRAS* (KRAS proto-oncogene, GTPase) and *TP53* (tumor protein p53) mutations had been detected in the cfDNA of healthy individuals [46]. In addition, Olbryt et al. performed the sequencing of formalin-fixed paraffin-embedded (FFPE) tumor and cfDNA samples derived from melanoma patients. The analysis revealed high concordance between the real-time quantitative PCR (qPCR) and NGS results of the *BRAF* (B-Raf proto-oncogene, serine/threonine kinase) mutation in FFPE samples (91%), as well as between the FFPE and cfDNA samples (91%) [47]. It is not only mutations of cfDNA that can be applied to cancer diagnosis, as fragment size of cfDNA [48,49], DNA methylation [24,50,51,52,53], and end coordinate [54,55] can also be used for the diagnosis of cancer. Mouliere et al. used differences in the length of cfDNA fragments to improve the sensitivity of detecting the presence of cfDNA and non-invasive genomic analysis of cancer [48]. Luo et al. found that a single ctDNA methylation marker, cg10673833, could yield high sensitivity (89.7%) and specificity (86.8%) for the detection of CRC and precancerous lesions in a high-risk population of 1493 participants [24]. *SEPT9* (septin 9) gene detection is the first U.S. Food and Drug Administration (FDA)-approved blood-based CRC screening test [56,57]. Studying the plasma cfDNA end characteristics in liver cancer patients, Jiang et al. found cancer-related end coordinates of cfDNA, which could be used for early diagnosis of cancer [55]. Cohen et al., developed a blood test called CancerSEEK based on cfDNA and circulatory protein biomarkers, which can detect 8 common cancers with a specificity of over 99% and a sensitivity of 69–98% (depending on the type of cancer) [58]. A combination of CancerSEEK and positron emission tomography-computed tomography (PET-CT) could reduce false positives to 0.4% [59]. These studies showed that cfDNA has great application value in the early diagnosis of cancer. Recently, a team developed a methylation-based method to analyze the “jagged ends” of cfDNA fragments. The results showed that the majority (87.8%) of cfDNA molecules were found to bear jagged ends. The average length of the jagged ends of fetal DNA molecules was longer than the average length of the mother, and the jagged ends of fetal DNA were generally tighter. In patients with liver cancer, tumor-derived DNA molecules showed more jagged ends than non-tumor DNA [60]. Our laboratory expanded cfDNA to the detection of open chromatin state [61,62]. Based on cfDNA, new epigenetic and genetic biomarkers were discovered to distinguish ESCA from normal people by using chromatin open state [62]. These studies open up new ideas for molecular diagnosis based on cfDNA in noninvasive detection.

### 2.2. Treatment Decisions and Prognosis

After the cancer is diagnosed, one can use the cancer biomarkers based on cfDNA to guide treatment, which greatly improves the treatment effect. Since the half-life of circulating cfDNA is between 16 min and 2.5 h [63], CfDNA can be used as a marker to reflect the overall changes in the disease [64]. This allows medical staff to monitor the treatment effect in real time and long-term for patients, so that scientific treatment adjustments and better prognosis can be made. In one prospective study, non-small cell lung cancer (NSCLC) patients with *EGFR* (epidermal growth factor receptor) exon 20 p.T790M positive mutations in plasma cfDNA had similar results as those treated with tissue tests using the *EGFR* inhibitor osimertinib (total response rates were 63% and 62%, respectively) [65]. To date, the FDA has approved the use of cfDNA for *EGFR* mutation detection to guide treatment of patients with NSCLC [66]. Many studies have shown that there is a correlation between total cfDNA levels and tumor stage (based on tumor size and degree of metastasis) [67,68], suggesting that cfDNA has prognostic ability. Moreover, the half-life of cfDNA is short, making it a real-time indicator of treatment effectiveness and may be observed earlier than clinical trials [39,69,70].

As is known to all, immunotherapy offers hope for about 30% of patients with advanced cancer, and unfortunately, clinicians do not know until treatment which patients are among the small group that will benefit. Lee et al. found that changes in the level of ctDNA released into the blood by the tumor during immunotherapy could predict the patient’s response to immunotherapy [71]. More recently, through the dynamic monitoring of ctDNA in patients with gastric cancer receiving immunotherapy, Jin et al. analyzed the correlation between ctDNA abundance and specific gene mutations and the efficacy of immunotherapy, and confirmed that the dynamic monitoring of ctDNA can indicate the efficacy of immunotherapy for gastric cancer, and in the analysis of drug resistance mechanism and the prediction of immune-related side effects, it also shows potential clinical value, providing a reference for the application of ctDNA dynamic monitoring in the immunotherapy of cancer [72]. We know that targeted therapies can place selective pressure on sensitive cancer cells, eventually leading to the evolution of cancer cells, leading to treatment resistance, and significantly reducing patient survival rate [42]. If the relative changes in cancer markers are observed early in the treatment process, doctors may be able to prepare a second treatment regimen to deal with newly developed drug-resistant cancer cells. The analysis of cfDNA is one way to constantly monitor changes in patients during treatment, such as mutations that make cancer cells resistant to drugs [73]. At present, the evidence for the role of cfDNA in monitoring treatment outcomes comes mainly from lung cancer, with the *EGFR* exon 20 p.T790M mutation leading to *EGFR* tyrosine kinase inhibitor resistance being reliably detected in the plasma cfDNA, 16 to 49 weeks before clinical or radiological progression is detected [74,75,76].

### 2.3. Minimal Residual Disease

Cancer is likely to recur even if it is successfully treated. One of the main challenges in cancer treatment is recurrence. Minimal residual disease (MRD) is a residual tumor component after therapeutic surgery or chemotherapy. The presence of MRD is a major cause of cancer recurrence. At present, MRD is difficult to be detected timely through imaging and biopsy. CfDNA can be used as a biomarker to detect MRD. Tie et al. [77] used massive parallel sequence analysis to assess the ability of ctDNA to detect MRD in plasma samples of removed CRC patients. Among the patients who did not receive adjuvant chemotherapy, 7.9% tested positive for ctDNA postoperatively, and 79% of those who tested positive for ctDNA had a recurrence after 27 months of follow-up. Only 9.8% of patients who tested negative for ctDNA relapsed. The presence of ctDNA in patients after chemotherapy was also associated with lower relapse-free survival. CfDNA detection after CRC resection provides direct evidence of MRD and identifies patients at high risk for recurrence. Therefore, cfDNA analysis of blood samples collected after surgery or after chemotherapy can identify patients at high risk of cancer recurrence, and thus modify or alter the management of treatment before large lesions develop.

## 3. Sequencing Techniques

CfDNA is highly fragmented DNA, and the percentage of ctDNA in total cfDNA is very low (in many cases <1.0%) [78]. Because of this, in the early stages of cancer development, we need detection techniques with better sensitivity and higher specificity to detect it, so that early treatment can be carried out and the survival rate of patients can be improved. However, the cost of high-sensitivity detection is generally more expensive, and it is not realistic to popularize it widely. For the detection and typing of advanced cancer, the concentration of ctDNA in patients with advanced cancer is much larger, so it has better sensitivity. Table 2 lists some available commercial platforms for ctDNA testing. These methods can be divided into two categories, targeted methods and non-targeted methods. The former requires detailed information on the tumor genome, with high detection sensitivity, including qPCR, digital PCR (dPCR) and targeted sequencing; the latter does not require prior knowledge of any specific cancer-related changes in the primary tumor, and usually uses the whole-genome or whole-exome sequencing; these methods are particularly important for discovering new cancer markers. Targeted methods and non-targeted methods can also be divided into three categories, qPCR-based, dPCR-based, and NGS-based.

### 3.1. qPCR-Based Technologies

Real-time quantitative PCR (qPCR) is the most widely used method in biological laboratories, with its simple operation and reliable results. It is a widely recognized gold standard. The qPCR method for detecting ctDNA is suitable for detecting known point mutations, such as therascreen *PIK3CA* RGQ PCR Kit (Qiagen, Hilden, Germany) [79], cobas^®^ *EGFR* Mutation Test v2 (Roche) [80], Target Selector™ *EGFR* Mutation Test Kit (Biocept, San Diego, CA, USA) [81] and Epi proColon^®^ (Epigenomics) [82,97]. The therascreen *PIK3CA* RGQ PCR Kit [79] is a real-time qualitative in vitro diagnostic PCR detection, which can detect 11 mutations of *PIK3CA* gene intissues or plasma of patients with breast cancer. It is the first companion diagnostic test approved by the FDA, which can be used to help select breast cancer patients who are suitable for treatment with the alpha-selective PI3K-inhibitor, alpelisib. The cobas^®^ *EGFR* Mutation Test v2 [80] is a qPCR test that identifies 42 mutations in exon 18, exon 19, exon 20 and exon 21 of the *EGFR* gene, including the exon 20 p.T790M resistant mutation. Target Selector™ *EGFR* Mutation Test Kit [81] detects *EGFR* mutations in DNA derived from plasma or FFPE tissue sections to give insight into cancer characteristics and provide biomarker status of tumors, such as NSCLC. Epi proColon^®^ [82] offers a convenient way of detecting CRC based on the methylation status of the *SEPT9* promoter in plasma cfDNA. Epi proColon is the first and only FDA-approved blood-based test for the detection of CRC. The test is available in the United States, Europe, China and selected other countries. The advantages of this type of method are simple operation and lower cost. However, it has its limitations. First, its sensitivity is relatively low. Second, it can only detect a limited number of gene loci. qPCR methods are limited in that they can detect only a few gene regions per reaction, and assays require >1% mutant allele fractions. Compared with the DNA analysis of cancer tissue samples, the test results of therascreen *PIK3CA* RGQ PCR Kit and cobas^®^ *EGFR* Mutation Test v2 have higher false negatives. Therefore, therascreen *PIK3CA* RGQ PCR Kit will be used for detection only when there are insufficient cancer tissue samples. In addition, patients whose test results are negative by cobas^®^ *EGFR* Mutation Test v2 need to undergo further routine biopsies. Therefore, it is very necessary to develop a more reliable, highly sensitive and highly specific detection method to replace the invasive tissue biopsy for the early diagnosis and monitoring of cancer.

### 3.2. dPCR-Based Technologies

The appearance of digital PCR (dPCR) plays an important role in ctDNA research. Compared with ordinary PCR, dPCR can directly count the number of DNA and realize the absolute quantification of samples. The detection sensitivity of dPCR has reached 0.01%, which is an ideal detection technique for mutant alleles in liquid biopsy samples [74]. For instance, the GENESTRAT^®^GENOMIC TEST (Biodesix) [83] is an on-market genomic test using dPCR to analyze cfDNA. GeneStrat delivers actionable, blood-based genomic test results within 72 h for patients with NSCLC. GeneStrat covers actionable mutations in the following genes: *EGFR* (exon 21 p.L858R, Del19, exon 18 p.G719X, exon 21 p.L861Q, exon 20 p.S768I, exon 20 p.T790M), *ALK*, *ROS1*, *RET*, *KRAS*, *BRAF*. The main drawback of the dPCR assay is that it can detect only one or several known mutations per reaction [98]. In addition to the efforts to improve sensitivity, it has been found that mutation detection at specific sites is insufficient, so there is an urgent need for technology that can quickly and accurately provide information at multiple specific sites, and NGS technology can precisely meet this need.

### 3.3. NGS-Based Technologies

Although PCR-based methods are highly sensitive and inexpensive to detect, they can only detect known variants, and the number of sites detected by these methods is limited. NGS technology overcomes these shortcomings. NGS has a high throughput feature and can screen for known or unknown variations. Currently, NGS can detect mutant allele fractions (MAF) of <1% [99]. NGS can be used in targeting panels to specifically and sensitively detect targeted ctDNA mutations [100].

CellMax Life’s liquid biopsy, CellMax-LBx [84], uses NGS to analyze 73 genes in the plasma ctDNA of patients to identify whether these genes have changed, so that the patient’s treatment effect can be monitored in real time, and clinical treatment can be better guided. CellMax-LBx liquid biopsy covers somatic gene alterations for solid tumors, as recommended by the National Comprehensive Cancer Network and the European Society of Medical Oncology, and that are associated with FDA-approved regimens and late-phase pharmaceutical clinical trials.

The Guardant360^®^ [85] assay is a breakthrough liquid biopsy based on cfDNA using NGS that provides comprehensive genomic profiling information, which can help patients with advanced cancer obtain the right treatment. The Guardant360^®^ test is also useful to pharmaceutical companies, as it is enabling the advancement of new therapies to the market faster. After obtaining a blood sample, Guardant360^®^ can provide comprehensive genomic results in about seven days in a laboratory, and the consistency with the tissue biopsy can reach 88.9%, so that patients can avoid the invasive risk of tissue biopsy and match the best treatment. Guardant360^®^CDx is the first FDA-approved blood test for complete genomic testing. A blood test does not require tissue testing, enabling more patients to benefit from the growing number of FDA-approved targeted therapies. Starting with the Guardant360^®^CDx test for complete genomic profiling identifies more patients with actionable biomarkers more quickly than starting with tissue biopsy. The test is also approved as a companion diagnostic to identify patients with NSCLC who may benefit from treatment with osimertinib, amivantamab-vmjw, and sotorasib. Since its launch in 2014, the Guardant360^®^ test has been ordered by more than 7000 clinicians more than 0.15 million times to help guide treatment for patients with advanced cancer.

The PGDx elio plasma resolve assay [89] is a non-invasive detection method based on patient plasma samples to detect 33 cancer-related genes. These genes have important roles both clinically and biologically. This method uses NGS technology to analyze ctDNA to assess whether they have undergone sequence mutation, gene amplification, translocation, and microsatellite instability. Combined with PGDx’s cancer genome analysis algorithms, this approach allows for the reliable detection and quantification of small fractions of tumor DNA in the plasma of individuals with cancer with high specificity and sensitivity (mutant allele fraction sensitivity ≥0.5%, depending on locus and alteration type).

The Circulating Cell-free Genome Atlas (CCGA) Study [93] is a predictive and observational longitudinal study based on NGS technology to characterize genomic cancer signals in the blood of cancer and non-cancer patients. The project has recruited more than 15,000 cancer patients and non-cancer patients. The plan is to follow these participants for at least five years to collect clinical data. The earlier the cancer is detected, the greater the chance of successful treatment. GRAIL and its research partners are recruiting CCGA participants to identify patterns that can be used to detect multiple cancers, and to discover, develop and validate blood tests for early detection of cancer. GRAIL also has five other liquid biopsy projects based on NGS and cfDNA, including the PATHFINDER Study [90], PATHFINDER 2 Study [91], SUMMIT Study [92], STRIVE Study [94] and the REFLECTION Study [95], for evaluating blood tests for early cancer detection. Recently, GRAIL announced the establishment of a partnership with the National Health Service (NHS) of the United Kingdom. It plans to provide British patients with Galleri™ (https://grail.com/galleri/, accessed on 12 May 2022), a blood test product for early screening of multiple cancers in 2021, to help improve the treatment of cancer patients. The commercial cooperation aims to confirm the clinical and economic performance of Galleri™ in the NHS system as a preliminary test for the NHS to routinely use the technology. Galleri™ is a blood test product based on cfDNA targeted methylation developed by GRAIL. It is expected to be launched in the United States in 2021 as a laboratory developed test (LDT) for cancer screening for asymptomatic people over 50 years of age. On March 2020, the GRAIL team announced the clinical validation data of the early version of Galleri™. The results showed that Galleri™ can distinguish more than 50 types of cancers at multiple stages through a single blood draw, including high mortality cancers and cancers that lack screening guidelines with a specificity of >99%, and a single false positive rate of less than 1%. When a cancer signal is detected, Galleri™ can also locate the tissue origin of the cancer with 93% accuracy. Currently, Galleri™ is being used in GRAIL’s first interventional study, PATHFINDER Study, in which Galleri™ will be used to guide clinical care. On June 2021, GRAIL announced the first data from PATHFINDER Study at the 2021 American society of clinical oncology (ASCO) Annual conference (https://grail.com/wp-content/uploads/2021/06/ASCO-2021-Pathfinder-Beer_FINAL-for-upload.pdf, accessed on 12 May 2022). At the same time, it was announced that the product will be available in the U.S. market, but it can only be used by doctor’s prescription as a supplement to the existing single-cancer screening methods. PATHFINDER 2 Study is also in progress. The enrollment of the PATHFINDER Study is about 6600, and the enrollment of PATHFINDER 2 Study is expected to reach more than 10,000. The follow-up time of PATHFINDER 2 Study has also been increased from 12 months in PATHFINDER Study to 3 years.

CfDNA can be combined with other cancer markers (e.g., proteins) for the early diagnosis of cancer. The PREEMPT CRC clinical study [96] of the Freenome company will recruit 14,000 participants between the ages of 45 and 85 to perform routine colonoscopy screening and take blood samples to verify an accurate and convenient CRC screening blood test. By decoding the complex cell-free biomarker model, Freenome’s blood tests are powered by their multigroup platform and designed to detect cancer in its early stages to help clinicians optimize the next generation of precision therapies. Freenome’s multi-group blood test approach combines cell-free cancer biology and machine learning to perform accurate early cancer screening by analyzing cfDNA, methylation, proteins and other biomarkers in plasma and decoding complex patterns associated with the body’s response to specific tumor types. At present, according to Freenome in the ASCO released gastrointestinal cancer symposium “Using multicomponent and machine learning for colorectal cancer early detection based on blood”(https://www.freenome.com/news-resources, accessed on 12 May 2022), the blood test has a sensitivity of 94% for the detection of early CRC. Freenome’s multi-component blood test was also compared with the leading fecal immunochemistry test (FIT), which showed a sensitivity of 100% for the multi-component blood test and 67% for the FIT. Therefore, a multi-component blood test is a worthwhile research direction of cancer early screening, which may improve the performance of cancer early screening.

## 4. Challenges

### 4.1. Detection and Analysis

CtDNA is not readily available in patients with early-stage tumors. In recent years, the research on ctDNA mainly focuses on the advanced cancer stage with high ctDNA content, while the research on the early cancer with low ctDNA content is lacking. The DNA extracted from the blood contains too much normal DNA, which has a great impact on the detection of ctDNA. CtDNA extraction lacks a standard. Because ctDNA is not very different from normal DNA, specific extraction is not very easy, and there is no standard for extraction. At present, most studies on ctDNA are conducted to extract the circulating DNA roughly through simple centrifugation or related kits, and then to determine ctDNA by sequencing for further analysis. It consumes a lot of manpower and material resources. Sequencing the entire loop of crude extracted DNA adds to the extra work and, at the same time, to the extra cost. In response to the problem of cfDNA sequencing, our laboratory developed SALP-seq (single strand adaptor library preparation-sequencing) [61,101]. SALP-seq has significant advantages in the construction of cfDNA NGS libraries. The adapted SALP-seq method can be used to prepare NGS libraries containing multiple cfDNA samples, which is useful for the efficient analysis of large clinical blood samples. Different samples can be labeled with different barcode T adaptors (BTAs). After the BTAs are connected, the final Illumina sequencing library is obtained by single-tube PCR amplification, and then the amplified libraries are mixed to obtain the final Illumina sequencing library, which improves the efficiency and reduces the cost.

Somatic mosaicism in plasma remains an immense challenge for the accurate interpretation of cfDNA liquid biopsy results [102]. Clonal hematopoiesis (CH) is part of the normal process of aging with the accumulation of somatic mutations and clonal expansion of hematopoietic stem cells [103]. The detection of these non-tumor derived CH-mutations has been repeatedly reported as a source of biological background noise of cfDNA liquid biopsy [102]. Incorrect classification of CH mutations as tumor-derived mutations could lead to inappropriate therapeutic management. The detection of mutations from plasma cfDNA analysis should be cautiously evaluated for their potential pathological relevance.

CtDNA NGS sequencing will generate a lot of data. Large amounts of data and complex data bring challenges to statistical analysis. Machine learning algorithms are expected to automate the diagnosis and detection of cancer-specific biomarkers, helping liquid biopsies. This may involve simple logistic regression or complex multi-layer artificial neural networks. In fact, machine learning has made some headway in liquid biopsies. For example, with machine learning, we can detect cancer with greater sensitivity and specificity [24,104,105]. However, the biggest shortcoming of machine learning algorithm is the lack of independent observation data. At present, the sample size of patients is generally in the tens to thousands, and based on the resolution of the base, a single patient may generate tens of billions of data. Ideally, machine learning algorithms should be set up with more of the former than the latter. Under the premise of insufficient data, over-fitting may occur in machine learning.

### 4.2. Clinical Applications

The development of detection and monitoring methods for cancer based on ctDNA biomarkers requires the study of large-scale clinical samples, not only to verify the effectiveness of the methods and the reliability of the biomarkers, but also to further verify the clinical practicality of the developed methods. For one type of cancer, hundreds or thousands of cancer patients may need to be analyzed. To investigate whether mutations can screen for cancer, the test should also assess the cfDNA of a large number of healthy individuals as a control. In addition, continuous clinical follow-up should be conducted to distinguish false positives from true positives.

To date, many liquid biopsy-based tests have been designed for the screening, diagnosis, and treatment guidance of cancer. Some of these tests are already commercially available for screening tests in cancer patients (Table 2). However, most studies on liquid biopsies are observational, and some lack healthy controls. Up to now, no studies have shown any improvement in patient outcomes or medical costs from liquid biopsies compared to standard monitoring [106]. In addition, few studies have evaluated the therapeutic efficacy based only on targeted therapies guided by ctDNA analysis. Few of the previous studies have focused on screening and early diagnosis of cancer. However, many large prospective studies are underway to rigorously demonstrate the clinical efficacy and usefulness of ctDNA testing. Guardant Health’s Shield™, for example, was a cfDNA-based test that was used to identify CRC at the earliest stages. In October 2019, Guardant Health launched an ECLIPSE trial to evaluate the performance of Shield™. This trial provides early screening for CRC by simply drawing blood. The study is expected to recruit about 10,000 people and, if successful, a marketing application will be submitted to the FDA (Table 2).

## 5. Conclusions and Future Perspectives

**Figure 2:**
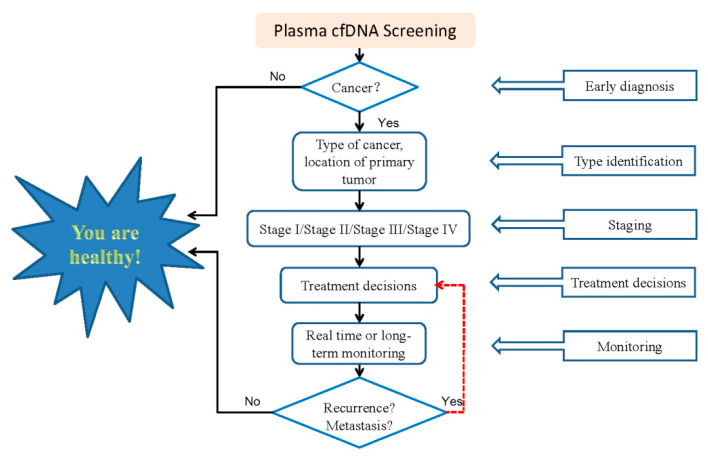
The workflow of cfDNA tests in clinical diagnosis, treatment and prognosis for cancer.

**Figure 3:**
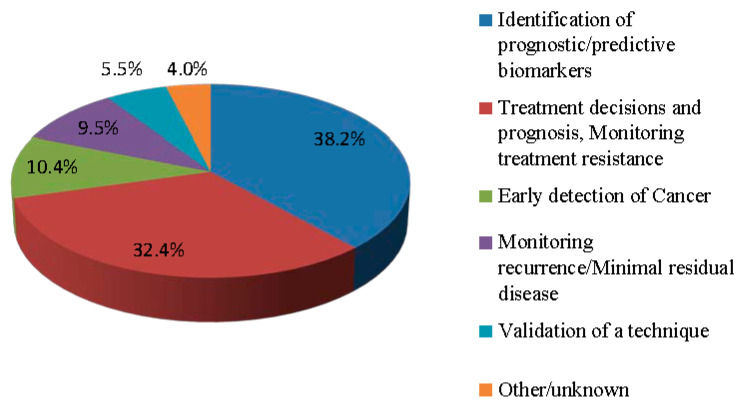
Current clinical trials using cfDNA in cancer settings. See the website (clinicaltrials.gov, accessed on 12 May 2022) for details of trials. Search performed on 12 May 2022.

As an analyte for liquid biopsy, cfDNA has been increasingly used in oncology. Figure 2 shows the workflow of cfDNA tests in the clinical diagnosis, treatment and prognosis for cancer. CfDNA screening can determine whether a person has cancer. If he/she is a cancer patient, what kind of cancer it is, where the lesion is, what stage of the cancer it is in, and what treatment method should be more reasonable. After treatment, the prognosis of patients can be observed and monitored in real time or long-term, based on cfDNA detection. In the case of recurrence or metastasis, through real-time monitoring of cfDNA, measures can also be taken in time for further treatment of the patient. Currently, more than 300 clinical trials are being conducted or actively recruited to investigate the diagnostic and prognostic marker utility of cfDNA incancers (Figure 3). However, we need to understand more about cfDNA. The focus in the future should be on sample collection, cfDNA isolation (increasing the yield of all relevant fragment sizes), and data analysis. Moreover, further research is needed to better understand the biological properties of ctDNA (e.g., the release and clearance mechanisms). At the same time, it is necessary to confirm the clinical validity and practicability of cfDNA as a biomarker for liquid biopsy, so as to further promote the clinical application of liquid biopsy.

As the consistency of detectable driving changes between cfDNA and solid biopsy increases, it is only a matter of time before this minimally invasive liquid biopsy becomes an important part of clinical and precision medicine. It is critical that only when the clinical effectiveness and clinical utility of cfDNA as a liquid biopsy biomarker are proven, it can exert its full potential and bring significant benefits to the clinical management of genome-driven oncology and cancer patients.

## Figures and Tables

**Table 1:** Comparison of applications between tissue biopsy and cfDNA.

	Tissue	cfDNA	Reference
Analysis of epigenetic alterations	+	+	[23,24,25]
Detection of mutations	+	+	[26,27,28,29]
Detection of copy number alterations	+	+	[30,31,32]
Potential for early detection of cancer	−	+	[33,34,35]
Potential for early detection of recurrence and Minimal residual disease(MRD)	−	+	[36,37,38]
Monitoring treatment response	−	+	[39,40,41]
Early identification of resistance mechanisms	−	+	[42,43,44]

“+” means possession of this ability, and “−” is the opposite.

**Table 2:** cfDNA liquid biopsy products.

Product/Study	Description	Company	Reference
Therascreen^®^*PIK3CA* RGQ PCR Kit	Accompanying diagnostic products for breast cancer to detect *PIK3CA* (phosphatidylinositol-4,5-bisphosphate 3-kinase catalytic subunit alpha) mutations in tissue and/or plasma ctDNA (liquid biopsy). Patients with negative liquid biopsy results should undergo tumor biopsy for *PIK3CA* mutation detection.	Qiagen	[79]
cobas^®^ *EGFR* Mutation Test v2	Detecting *EGFR* mutations in plasma cfDNA from patients with lung cancer. Guiding decision therapy. FDA-approved.	Roche	[80]
Target Selector™ *EGFR* Mutation Test Kit	Detecting *EGFR* mutations in DNA derived from blood plasma or FFPE tissue sections to give insight into cancer characteristics and provide biomarker status of tumors, such as NSCLC.	Biocept	[81]
Epi proColon^®^	Offering a convenient way of detecting CRC based on the methylation status of the *SEPT9* promoter in plasma cfDNA. FDA-approved.	Epigenomics	[82]
GENESTRAT^®^GENOMIC TEST	Providing blood-based mutation results of *EGFR*, *ALK* (ALK receptor tyrosine kinase), *ROS1* (ROS proto-oncogene 1, receptor tyrosine kinase), *RET* (ret proto-oncogene), *BRAF*, and *KRAS* for lung cancer diagnosis.	Biodesix	[83]
CellMax-LBx	Using a routine blood sample to profile 73 genes from ctDNA to identify and assess actionable genomic alterations. CellMax-LBx liquid biopsy genetically analyzes almost all cancer types. Targets treatments, tracks responses and monitors recurrence.	CellMaxLife	[84]
Guardant360^®^	Providing fast, accurate and comprehensive genomic results from a simple blood draw to help patients with advanced cancer choose treatment based on changes in ctDNA detected in different solid tumors.	Guardant Health	[85]
InVisionFirst^®^-Lung	Testing 37 genes relevant to the care of patients with advanced NSCLC based on ctDNA NGS liquid biopsy.	Inivata	[86]
Shield™	Using cfDNA-based test to identify CRC at the earliest stages.	Guardant Health	[87]
Guardant Reveal™	The first blood-only test that detects residual and recurrent disease, without the need for a tissue biopsy. Detecting ctDNA in blood after surgery to identify patients with residual disease who may benefit most from adjuvant therapy. The first indication is early-stage CRC with additional cancer types to follow.	Guardant Health	[88]
PGDx elio™plasma resolve	Providing blood-based mutation results of 33 gene panels for cancer diagnosis.	Personal Genome Diagnostics (PGDx)	[89]
PATHFINDER Study	Evaluating a blood test for the early detection of multiple cancer types.	Grail	[90]
PATHFINDER 2 Study	Evaluating a blood test for the early detection of multiple cancer types.	Grail	[91]
SUMMIT Study	Evaluating a blood test for the early detection of lung cancer.	Grail	[92]
Circulating Cell-free Genome Atlas (CCGA) Study	Evaluating a blood test for the early detection of cancer.	Grail	[93]
The STRIVE Study	Evaluating a blood test for the early detection of breast cancer.	Grail	[94]
REFLECTION Study	Understanding the performance of Galleri^®^ test in clinical settings and the impact on patients and healthcare providers.	Grail	[95]
PREEMPT CRC clinical study	Evaluating a blood-based test for the early detection of colorectal cancer.	Freenome	[96]
